# Task Complexity and Image Clarity Facilitate Motor and Visuo-Motor Activities in Mirror Therapy in Post-stroke Patients

**DOI:** 10.3389/fneur.2021.722846

**Published:** 2021-09-22

**Authors:** Umar Muhammad Bello, Chetwyn C. H. Chan, Stanley John Winser

**Affiliations:** ^1^Department of Rehabilitation Sciences, The Hong Kong Polytechnic University, Kowloon, Hong Kong, SAR China; ^2^Department of Physiotherapy, Yobe State University Teaching Hospital, Damaturu, Nigeria; ^3^Department of Psychology, The Education University of Hong Kong, Tai Po, Hong Kong, SAR China

**Keywords:** post-stroke, mirror therapy, movement complexity, image clarity, primary motor cortex, precuneus

## Abstract

**Introduction:** Mirror therapy is effective in the recovery of upper-limb function among post-stroke patients. An important component of mirror therapy is imagining finger movements. This study aimed to determine the influence of finger movement complexity and mirror image clarity on facilitating motor and visuo-motor activities in post-stroke patients.

**Methods:** Fifteen post-stroke patients and 18 right-handed healthy participants performed simple or complex finger tapping while viewing mirror images of these movements at varying levels of clarity. The physical setup was identical to typical mirror therapy. Functional near infrared spectroscopy (fNIRS) was used to capture the brain activities elicited in the bilateral primary motor cortices (M1) and the precuneus using a block experimental design.

**Results:** In both study groups, the “complex finger-tapping task with blurred mirror image” condition resulted in lower intensity (*p* < 0.01) and authenticity (*p* < 0.01) of the kinesthetic mirror illusion, and higher levels of perceived effort in generating the illusion (*p* < 0.01), relative to the “simple finger-tapping with clear mirror image” condition. Greater changes in the oxygenated hemoglobin (HbO) concentration were recorded at the ipsilesional and ipsilateral M1 in the “complex finger-tapping task with blurred mirror image” condition relative to that recorded in the “simple finger-tapping task with clear mirror image” condition (*p* = 0.03). These HbO concentration changes were not significant in the precuneus. Post-stroke patients showed greater changes than their healthy counterparts at the ipsilesional M1 (*F* = 5.08; *p* = 0.03; partial eta squared = 0.14) and the precuneus (*F* = 7.71; *p* < 0.01; partial eta squared = 0.20).

**Conclusion:** The complexity and image clarity of the finger movements increased the neural activities in the ipsilesional motor cortex in the post-stroke patients. These findings suggest plausible roles for top-down attention and working memory in the treatment effects of mirror therapy. Future research can aim to corroborate these findings by using a longitudinal design to examine the use of mirror therapy to promote upper limb motor recovery in post-stroke patients.

## Introduction

A plane mirror is a simple tool used daily to provide instant visual feedback on body appearance, thereby influencing self-recognition (1). Due to these properties, the plane mirror has been used in rehabilitation clinics as a biofeedback apparatus for balance and postural training (2). Advanced therapeutic application of a plane mirror is an essential element of Ramachandran's mirror therapy or the mirror-induced visual illusion (MVI) paradigm (3).

Mirror therapy has been widely used as a rehabilitative intervention to enhance motor performance in post-stroke patients (4, 5). Moderate evidence supports the effect of mirror therapy for the regain of motor function of upper and lower limbs in this population (5–7). The key feature of mirror therapy is viewing images of motor activity of the unaffected limb to generate an illusion of moving the hidden, affected limb. The generation of the mirror illusion has been associated with activations in the primary motor cortex (M1), precuneus, premotor cortex, primary somatosensory cortex, cerebellum, dorsolateral pre-frontal cortex, superior temporal gyrus and posterior cingulate cortex (8–11), which subserve motor, cognitive and perceptual processes (8, 12). Long-term training using mirror therapy leads to upward regulation of activity in the ipsilesional M1 in post-stroke patients (13), which contributes to post-stroke patients' upper-limb functional regain (14).

Recent studies conducted by our research team highlighted mental imagery as one potential explanation for the useful changes in upper limb function during mirror therapy due to consistent overlap of neural activity reported in the M1, precuneus, primary somatosensory cortex, and cerebellum (10, 11). Another theory explaining the mental processes of mirror therapy in the literature is the mirror neuron system theory (15, 16). However, the theory is limited in substantiating some of the basic neural mechanisms of the mirror therapy paradigm [see Bello et al. (10) and Bello et al. (11)]. For example, lack of significant number of neural substrates of the mirror neuron system, found sub-serving the mirror therapy mental procesess (8), and the activation of ipsilateral M1 in the mirror therapy paradigm, while viewing an immobile image of a hand holding a pencil (17, 18). On the other hand, “Mental imagery” which denotes the rehearsal of limb movement without actual execution (19, 20), shares overlapping neural substrates and associated mental processes with mirror therapy. It can further be classified into kinesthetic motor imagery and visual motor imagery, with the former yielding greater activity in the motor areas and inferior parietal lobule (21, 22).

Briefly, mental imagery theory postulates that the mirror therapy paradigm generates visual image of an “imagined action” of the hidden static limb, owing to the similarities between the hidden limb and the mirror inverted image (23, 24). Kinaesthetic motor imagery, as a modality of mental imagery is associated with the internal generation of sensory components of movement (for example kinesthesia, muscle stretching and joint mobility), associated with the execution of similar movement (20, 22). Existing literature indicated that kinaesthetic motor imagery evokes activation of the motor system without visible mobility of the imagined body part (22, 25, 26). This could serves as the basis for the presence of kinaesthetic mirror illusion (27, 28) in the mirror therapy paradigm (10).

### Rationle for the Hypotheses

Kinaesthetic motor imagery [which is closely associated with mirror therapy (10)] is influenced by the complexity of the mentally rehearsed motor sequences (19, 29, 30). Rehearsing complex finger movements showed an increase in activation of the premotor cortex, supplementary motor area, posterior parietal and cerebellar regions (19), and higher motor-evoked potential (MEP) amplitude than rehearsing simpler finger movements (19, 30). Increased sequence length also increased neural activity in the premotor cortex, superior parietal areas and the cerebellar vermix (29). The increased brain activation when rehearsing more complex or longer tapping sequences reflects a possible increase in cognitive load (working-memory, attention, and motor-related processes) relative to rehearsing simpler and shorter tapping sequences (31–33). A motor sequence requires the execution of preprogrammed movement patterns, which requires the premotor and supplementary motor areas to generate the sequence from memory and fit it into a precise timing plan (34). Moreover, dissociable aspects of cognitive demands may include encoding and retrieval of information, kinesthesia, motor preparation and movement selection (31, 32). Specifically, increased activity in the premotor and supplementary motor areas, and associated corticospinal excitability (MEP), has been found during more complex kinesthetic motor imagery of finger movements. This increased activity is not found during simpler imagery processes, and is thus indicative of a “task-dependent intracortical facilitatory effect on M1 during imagery” (19). In addition to complexity, visual information load is another factor found to increase cognitive load during mental imagery (35) due to the overlap of the mental processes of visual attention/visual working memory with mental imagery (36). With limited attention, recognition and memory are both reserved (37), as in the case of mental rehearsal of blurred images, thereby degrading the image quality and increasing the information load (35). Similarly, behavioral studies found that lower clarity of the mirror image reduced the kinesthetic mirror illusion (38).

As cognitive load during mental imagery is influenced by task sequence and image quality, we examine the influence of these two factors on the effect of mirror therapy, given the overlap in the neural processing between the two techniques (8, 10, 11). This study investigates how finger movement complexity and image clarity in the mirror therapy paradigm influence associated motor responses and visuo-motor memory in post-stroke patients and healthy participants. Changes in neural activities in the ipsilesional and ipsilateral M1 and precuneus, which are common parameters in this regard, were examined. Motor-related neural activities were captured with fNIRS. Firstly, based on the findings reported in the previous paragraph regarding the influence of increased sequence length and visual information load on cognitive load, we hypothesized that a complex finger-tapping sequence and a blurred image would result in stronger ipsilesional/ipsilateral top-down motor activity facilitation, reflected by increased blood oxygenated hemoglobin concentration in the ipsilesional/ipsilateral M1, relative to a simple finger tapping sequence and a clear image. Secondly, due to the reduced extent of ipsilesional neural activity and an associated decline in the inter-hemispheric activation balance among post-stroke patients (14, 39), it is expected that, in comparison with healthy controls, this group of patients would respond differentially to the various manipulations in the mirror therapy paradigm.

## Materials and Methods

### Participants

This study included 15 post-stroke patients (8 patients with left hemiplegia, 11 males, mean age: 60.9 ± 6.8 years old) and 18 healthy participants (10 males, mean age: 61.1 ± 7.4 years old). Participants were recruited by convenience sampling. Inclusion criteria of the post-stroke patients were: (1) right-handed male or female adults, (2) aged 40–75 years with normal or corrected-to-normal vision and hearing, and (3) without a history of severe deficits in memory, communication and understanding of verbal instructions Mini-Mental State Examination >24 points (40). Exclusion criteria for post-stroke patients were: (1) recurrent stroke and/or trauma affecting voluntary movement of the unaffected upper extremity. Inclusion criteria for healthy participants were: (1) right-handed male or female adults, (2) 40–75 years old with normal or corrected-to-normal vision and hearing, and (3) without a history of psychiatric or neurological disorders. Sample size was calculated using the G-power software (http://www.gpower.hhu.de/): based on 90% power, 5% type I error and medium partial eta-squared (η^2^) of 0.06, which indicated that a sample size of 15 participants in each group was needed to detect significant within- and between-group differences in change in oxygenated hemoglobin concentration (HbO) in the M1 and precuneus. Ethical approval was obtained from the human subjects' ethics sub-committee of the Hong Kong Polytechnic University before the study commenced (HSEARS20190129002). The study was registered with the Hong Kong University Clinical Trial Registry (HKUCTR-2754).

### Experimental Setup

**Figure 1:**
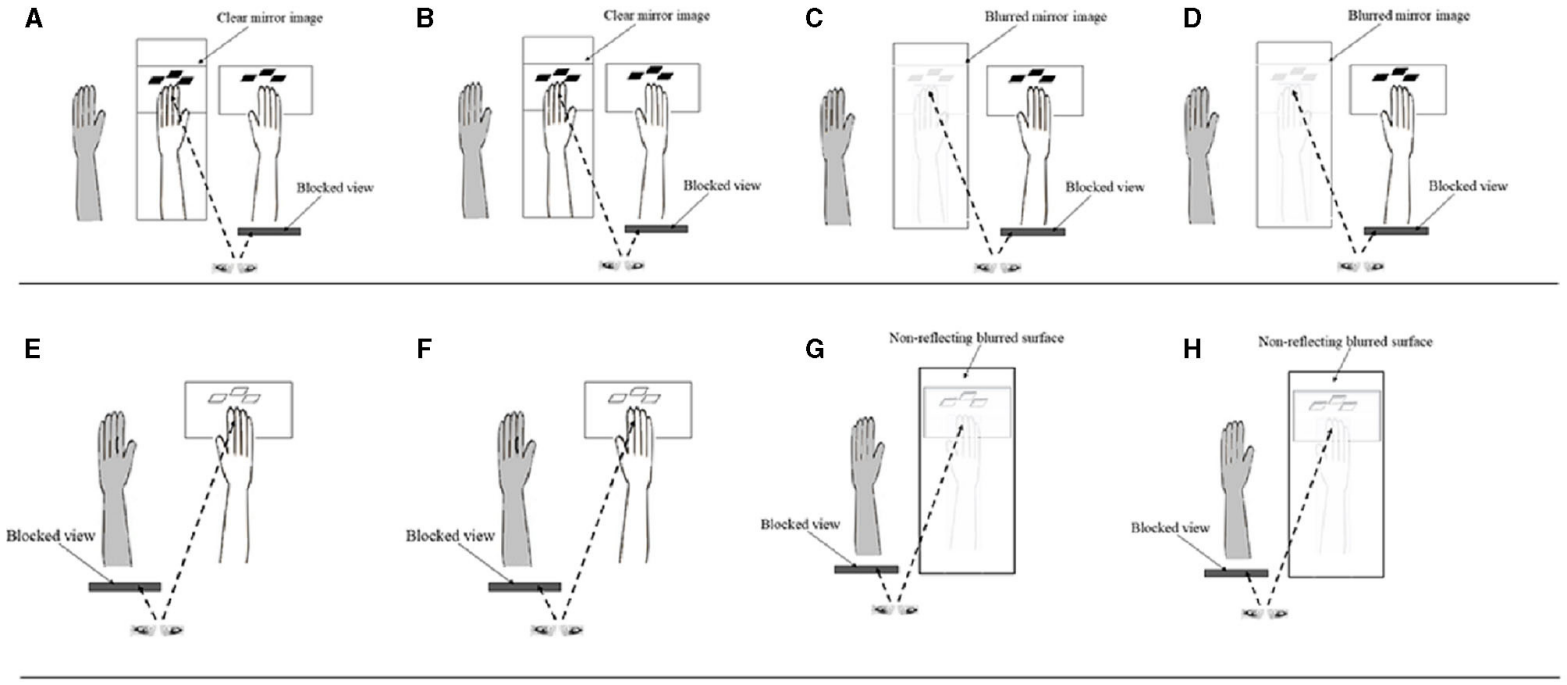
Schematic diagrams of the different conditions in the study. **(A)** Mirror Clear + Simple; **(B)** Mirror Clear + Complex; **(C)** Mirror Blurred + Simple; **(D)** Mirror Blurred + Complex; **(E)** Direct Clear + Simple; **(F)** Direct Clear + Complex; **(G)** Direct Blurred + Simple; and **(H)** Direct Blurred + Complex (Control conditions: **E–H**). Ash colored limb depicts the static affected or left limb (among healthy participants), hidden behind the mirror (in panels **A–D**).

Detailed methods used in this study have been published elsewhere (41). The plane mirror setup was placed on a table in a well-lit and quiet room. Each participant was seated comfortably at the table on which a mirror (30 × 24 inches) was placed at a 90° angle facing the left or right side (depending on right or left hemiplegia of patient participants) of the table surface corresponding to the mid-sagittal plane of the participant (see Figure 1A). The distance of the mirror was about 10 cm from the midline. Participants placed their affected (for patients) or left forearm/hand (for healthy participants) 15 cm behind the erected mirror, while the other hand rested on the table in front of the reflecting surface of the mirror. Participants were instructed to perform finger tapping strokes with their unaffected or right hand on a wireless keyboard (Mofii X210, Shenzhen SQT Electronics Co., Ltd.) with index, middle and ring fingers. During the tapping, participants were required to observe images of the moving fingers projected onto the mirror while the affected or left forearm/hand remained stationary. The frequency of the finger tapping movements was paced at 1.5 Hz (90 b/m) using an auditory metronome. Participants were instructed to view the movement images presented on the mirror as their own moving forearm/hand (42, 43), as well as to sense the kinesthetic sensation associated with the movements presented by the images.

### Task Design

This study used a 2 × 2 block design that manipulated finger tapping complexity (simple vs. complex) and mirror image clarity (clear vs. blurred), resulting in four experimental conditions (Figures 1A–D). Each condition had a separate control condition, in which participants were instructed to directly view the movements of their forearm/hand without the mirror (Figures 1E–H). We used the control conditions to elicit the ipsilateral excitations to contrast with those resulting from the experimental conditions (44). There was a total of eight conditions, with each condition consisting of 10 blocks of movement cycles (Table 1). Each block lasted for 20 s, during which participants tapped their fingers cyclically and rhythmically according to the prescribed complexity at a frequency of 1.5 Hz (45). The beginning and the end of each block were indicated by a sound generated from E-prime software. All finger movements were performed by the unaffected (for post-stroke patients) or right hand (for healthy participants) index, middle and ring fingers, which tapped the red, pink, and green keys on a keyboard, respectively. Simple and complex tapping cycle conditions involved the index, middle and ring fingers, making three sequential and nine non-sequential taps, respectively (Table 1). Clear and blurred image clarity conditions involved the participants viewing movement images from a clear or 35% blurred (with a mesh) mirror. After completing one block, participants paused for 20 s before commencing the next block of the tapping cycle until the end of the assessment condition (46). After completing each of the four finger tapping conditions with the mirror visual feedback, participants were asked to respond to three questions on the intensity and authenticity of the kinesthetic mirror illusion, and the perceived effort used to generate the kinesthetic sensation. Similarly, checks were conducted to authenticate participants' attention on the finger movements across the assessment conditions by asking intermittent question on the color of the first or last key pressed during the finger tapping. The sequence of the eight conditions was randomized for each participant using E-prime to control for possible order effects (46). Each participant completed 80 blocks, which took about 74 min.

**Table 1:** Descriptions of different finger tapping conditions in terms of task complexity and contents.

**Conditions**	**Task complexity**	**Task contents (all finger tapping paced at 1.5 Hz)**
Mirror Clear + simple	Simple finger tapping movement	Repeated tapping of colored keys with the index, middle and ring fingers + clear mirror visual feedback
Direct Clear + simple (control condition)	Simple finger tapping movement	Repeated tapping of colored keys with the index, middle and ring fingers + direct view of clear actively moved hand
Mirror Clear + complex	Complex finger tapping movement	Tapping sequence: ring × 2, index × 2, middle × 2, ring × 1, middle × 1 and index × 1 on colored keys + clear mirror visual feedback
Direct Clear + complex (control condition)	Complex finger tapping movement	Tapping sequence: ring × 2, index × 2, middle × 2, ring × 1, middle × 1 and index × 1 on colored keys + direct view of clear actively moved hand
Mirror Blurred + simple	Simple finger tapping movement	Repeated tapping of colored keys with the index, middle and ring fingers + blurred mirror visual feedback
Direct Blurred + simple (control condition)	Simple finger tapping movement	Repeated tapping of colored keys with the index, middle and ring fingers + direct view of blurred actively moved hand
Mirror Blurred + complex	Complex finger tapping movement	Tapping sequence: ring × 2, index × 2, middle × 2, ring × 1, middle × 1 and index × 1 on colored keys + blurred mirror visual feedback
Direct Blurred + complex (control condition)	Complex finger tapping movement	Tapping sequence: ring × 2, index × 2, middle × 2, ring × 1, middle × 1 and index × 1 on colored keys + direct view of blurred actively moved hand

### Finger Tapping Training

Before the experiment, participants completed a 1-h practice session to familiarize themselves with the simple and complex finger-tapping sequences. Participants were instructed to synchronize each tapping stroke with a beat of the metronome set at a frequency of 1.5 Hz. The practice continued until participants reached an accuracy rate of 90% on all tapping sequences.

### Behavioral and Clinical Assessment

A finger tap error was defined as any tap that did not occur in correspondence to a prescribed simple or complex sequence. The error rate was calculated as the “total number of wrong taps/total number of taps” (47). The taps made by participants on the three colored keys of the wireless keyboard were transmitted to a notebook computer throughout each task condition. The sequence of the taps made by the participants was matched with the prescribed sequence, based on which the error rate was computed for each task condition.

After completing each of the four experimental conditions with mirror visual feedback, participants were asked to name the color of the first or last key tapped to ensure active task participation. The responses were documented as either correct or incorrect.

Similarly, questions on the intensity and authenticity of the kinesthetic mirror illusion were asked using a questionnaire [Appendix A in Supplementary Material; modified from Diers et al. (48) and Roberts et al. (49)] after completing the tasks for each of the four experimental conditions. Question items on intensity (*To what extent did you feel that the movement of the displayed hand belonged to your affected/left hand?*) and authenticity (*To what extent did you feel as you were seeing your real hand?*) of the illusion were adopted from Diers et al. (48). Lower scores were interpreted as higher intensity and authenticity of the kinesthetic mirror illusion. The rating parameters of the clarity of the items (1 = *perfectly clear and as vivid as normal felt movement*, 2 = *clear and reasonably vivid*, 3 = *moderately clear and vivid*, 4 = *vague and dim*, 5 = *not felt at all*) were adopted from Roberts et al. (49). Furthermore, a question assessing perceived effort in generating the kinesthetic sensation of the left/affected hand (question 3) was asked. A rating scale of 1 (*very much effort*) to 5 (*no effort at all*) was utilized to answer question 3, with lower scores indicating greater effort in generating the kinesthetic sensation.

The Mini-Mental State Examination (MMSE) (50), Modified Ashworth Scale (MAS; elbow, wrist and finger) (51) and Fugl-Meyer Motor Assessment (FMA; wrist and hand) (52) were used to assess cognition, spasticity, and upper limb function, respectively, among the post-stroke patients. The MMSE includes 20 tests categorized into 11 domains, including orientation (time: 5 points; place: 5 points), attention (5 points), registration memory (3 points), recall (3 points), naming (2 points), repetition (1 point), following three-stage command (3 points), reading (1 point) writing (1 point), and copying intersecting pentagons (1 point) (53). Higher MMSE scores indicate better cognitive ability, with overall scores ranging from 0 to 30 points.

The elbow, wrist and finger sub-scales of the MAS were used in this study. The level of spasticity in each of the muscle group(s) was assessed using a 6-point rating scale, with higher scores indicating a greater level of spasticity (51). The wrist and hand sub-scales of the FMA were utilized to examine the upper limb function of post-stroke patients. Items in the sub-scales were rated using a 3-point ordinal scale. The overall scoring for wrist and hand sub-scales ranges from 0 to 10 and 0 to 14 points, respectively (52), with a higher score indicating better wrist and hand function.

### fNIRS Recordings and Regions of Interest (ROIs)

fNIRS was used to capture the neurovascular changes, assessed as HbO, in the bilateral M1 and precuneus when participants performed the finger tapping tasks (46). We used the fNIRS in line with previous similar mirror therapy studies (54–56). Importantly, fNIRS has been found to have a higher tolerance of interferences caused by a participant's postural movements when compared to other methods, such as functional magnetic resonance imaging (57). The fNIRS setup consisted of 18 emitters and 16 detectors (ETG-4000, Hitachi Medical Co., Tokyo, Japan). Cap mounting and placements of the optodes (3-by-3 and 3-by-5 probe sets) on the ROIs (bilateral M1 and bilateral precuneus) followed those reported by Mehnert et al. (55). The method to locate the Cz, C3, and C4 positions on the surface of the scalp followed the steps described by Jurcak et al. (58). The left and right M1 corresponded to the locations of C3 and C4, respectively. The total areas of coverage of the scalp were defined by the 3 cm distances between the emitters and detectors on each of the left and right hemispheres.

### fNIRS Data Preprocessing

Data processing was performed using the MATLAB toolbox HomER2 (59). Firstly, optical density change was generated from the data on raw intensity (60), and the motion artifacts were then corrected using the Spline interpolation algorithm (61). Secondly, a band-pass filter of 0.01–0.2 Hz was used to further process the optical density changes (62). Thirdly, optical density was then converted to changes of HbO and deoxygenated hemoglobin (Hb) concentrations at different time points using the Beer-Lambert law (60). Mean changes in HbO and Hb were computed for each participant for each task condition. For testing the between-group and between-condition differences, changes in HbO was used due to its higher sensitivity in detecting cortical regional blood flow (63).

### Statistical Analysis

Between-group differences in socio-demographic characteristics and a validity check of task participation were compared using an independent *t*-test, the Mann-Whitney *U*-test, and chi-square statistics. An independent *t*-test was used to examine the differences between the patients with left and right hemiplegia among post-stroke patients, in terms of socio-demographic and clinical measures. The mirror effect (ME) was computed by subtracting the mean changes in HbO of control conditions from their corresponding experimental conditions [ME: (MVF > DVAH)]. A mixed model analysis of variance (ANOVA) was conducted to assess the influence of finger tapping complexity and mirror image clarity on the HbO of the ipsilesional/ipsilateral M1 and the precuneus. *Post-hoc* analysis with the Bonferroni correction was conducted to examine pairwise differences across the experimental conditions. A mixed model ANOVA was used to test the within-between differences in participants' response to the varied intensity and authenticity of the kinesthetic mirror illusion and perceived effort in generating kinesthetic sensation. Statistical significance was set at *p* ≤ 0.05. All data analyses were conducted using the Statistical Package for Social Sciences software (SPSS, version 26, IBM, Armonk, NY, USA).

## Results

### Socio-Demographic and Clinical Assessment Results

No significant difference was found in age for post-stroke patients (Mean ± SD: 60.9 ± 6.8) and healthy participants (61.1 ± 7.4); *t*(31) = −0.10, *p* = 0.92 (Table 2 and Appendix B in Supplementary Material). Similar results were found for educational levels of the different study groups, *U* = 120.5, *p* = 0.61. No significant association regarding gender distribution was found between the study groups χ^2^(1) = 1.76, *p* = 0.19. Among the post-stroke participants, the mean age of disease onset was 60.6 ± 37.0 months and nine of 15 (60%) had a haemorrhagic stroke. The majority of the patient participants (53.3%) were having left-sided hemiplegia. No significant differences were found between the results obtained from patients with left and right hemiplegia in terms of age (*t* = −0.51, *p* = 0.62), average period of stroke onset (*t* = 0.87, *p* = 0.40), MMSE (*t* = −0.66, *p* = 0.52), MAS-elbow (*t* = 0.20, *p* = 0.85), MAS-wrist (*t* = −0.88, *p* = 0.39), MAS-finger (*t* = −1.15, *p* = 0.27), FMA-wrist (*t* = 0.29, *p* = 0.80) and FMA-hand (*t* = 0.65, *p* = 0.52).

**Table 2:** Socio-demographic and clinical characteristics of the post-stroke patients (*n* = 15).

**Patients**	**Age (yrs)**	**Sex**	**Edu. level**	**Lesion**	**Affected side**	**Time sincestroke (months)**	**Etiology**	**Use ofvisual aid**	**MMSE**	**MASelbow**	**MAS wrist**	**MASfinger**	**FMA wrist**	**FMA hand**
1	52	M	Sec	NA	L	51	H	Yes	30	1	0	0	6	6
2	65	M	Sec	NA	R	89	I	Yes	27	3	3	3	2	0
3	60	F	Sec	NA	L	92	I	Yes	29	0	0	0	10	14
4	60	M	Pri	NA	R	66	H	No	30	0	0	0	10	14
5	61	M	Deg	NA	R	28	I	Yes	30	0	0	0	10	14
6	47	F	Sec	Left Lentiform nucleus	R	37	I	Yes	30	1	1	1	7	9
7	68	F	Pri	NA	L	7	H	Yes	27	2	1	1	5	10
8	60	M	Deg	NA	L	50	H	Yes	29	2	1	0	10	12
9	55	M	Sec	NA	L	105	H	Yes	26	2	1	1	7	8
10	60	M	MSc	Basal ganglia	L	47	H	Yes	30	0	0	0	10	14
11	75	F	Sec	Right Pontine infarct	L	33	I	Yes	30	0	0	0	10	14
12	64	M	Pri	Corona radiata	L	23	I	No	29	0	1	1	8	7
13	68	M	Sec	Left Frontal lobar hemorrhage	R	149	H	Yes	29	0	0	0	10	14
14	61	M	Sec	NA	R	54	H	Yes	27	0	0	0	10	13
15	57	M	Sec	NA	R	78	H	No	30	0	1	1	6	9
	60.9 ± 6.8	M:73.3%			L:53.3%	60.6 ± 37	H:60%	Yes:80%	28.9 ± 1.4	0.7 ± 1	0.6 ± 0.8	0.5 ± 0.8	8 ± 2.5	10.5 ± 4.1

*NA, not available; yrs, years; MMSE, Mini mental state examination; MAS, Modified Ashworth scale; FMA, Fugl-meyer motor assessment; M, male; F, female; Sec, secondary; Pri, primary; Deg, first degree; R, right; L, left; H, haemorrhagic stroke and I, ischaemic stroke*.

*Maximum scores of MMSE: 30 points; MAS elbow, wrist and finger: 4; FMA wrist: 10; FMA hand: 14*.

*Hand dominance: All the participants were right handers*.

### Mean Change of HbO in M1 and Precuneus Across Experimental and Control Conditions

Mean changes (in contrast with baseline) of HbO in the M1 and Precuneus across the experimental and control conditions among the patient and healthy participants are summarized in Appendix C in Supplementary Material.

### Mirror Effect Changes in HbO at Ipsilesional/Ipsilateral M1

**Figure 2:**
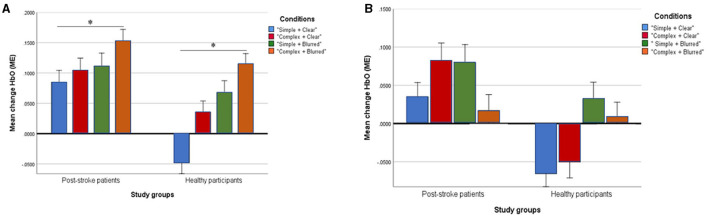
Profile plots showing changes of mean [HbO] (ME) in ipsilesional/ipsilateral M1 **(A)** and contralesional/contralateral M1 **(B)** in four experimental conditions between post-stroke patient and healthy groups (unit, μMol/L). *Signify statistical significance.

**Figure 3:**
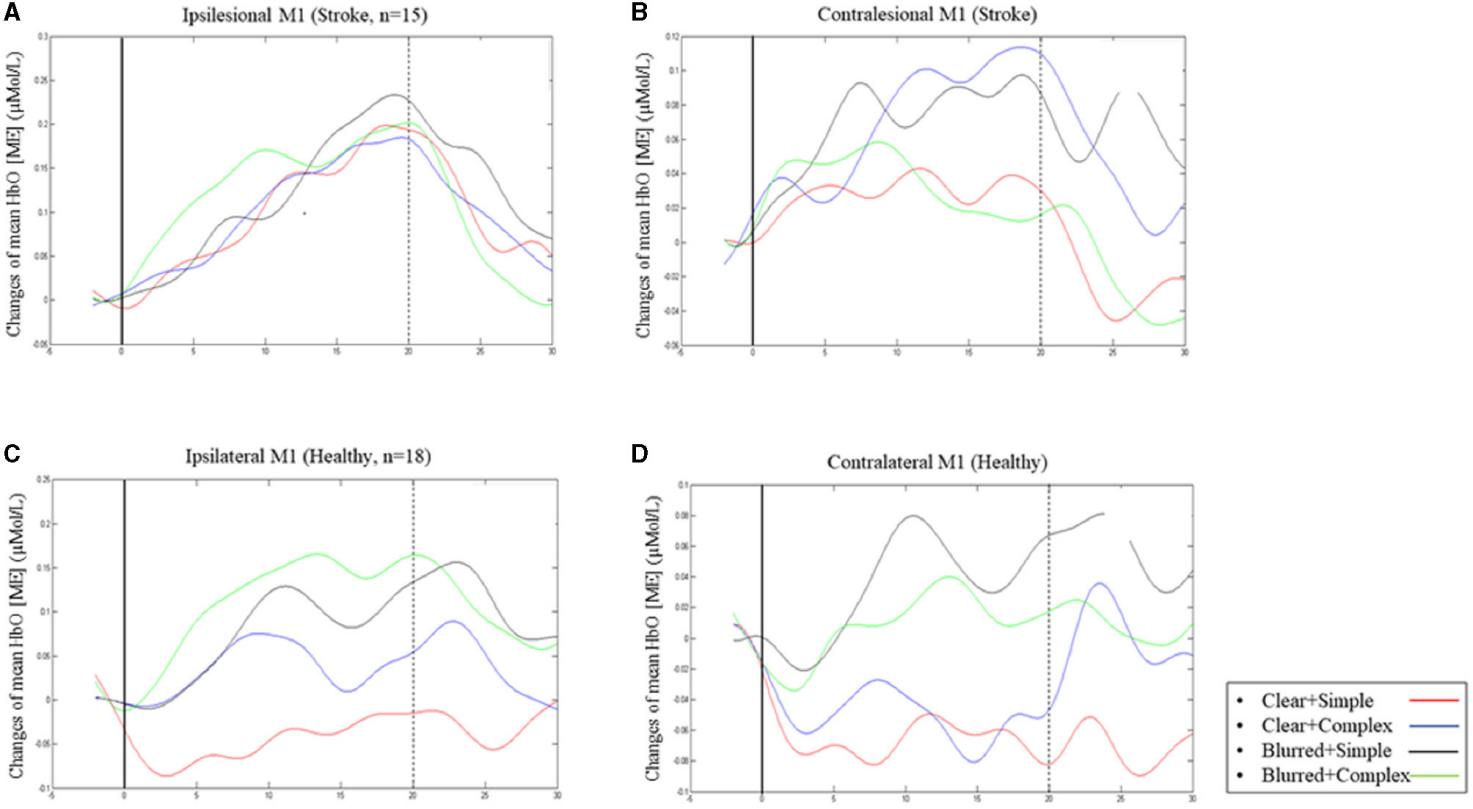
Changes of mean [HbO] (ME) across 20 s finger tapping in ipsilesional M1 **(A)** and contralesional M1 **(B)** in patient group; changes in mean [HbO] (ME) in ipsilateral M1 **(C)** and contralateral M1 **(D)** in healthy group.

The group effect on HbO was statistically significant [*F* = 5.08; *p* = 0.03; partial eta squared = 0.14], with patient participants having significantly higher mean values than healthy adults across all experimental conditions (Table 3). The condition effect was also significant [*F* = 3.53; *p* = 0.02; partial eta squared = 0.10], but the group × condition effect was not statistically significant [*F* = 0.72; *p* = 0.54, partial eta squared = 0.02]. A *post-hoc* pairwise analysis on condition effects revealed significantly higher changes in HbO (*p* = 0.03) for the “complex and blurred” condition relative to the “simple and clear” condition; no other pairwise differences were significant (Figure 2A). However, a trend was observed of greater changes in HbO in the complex sequence and the 35% blurred sequence relative to the “simple and clear” condition for both patient and healthy participants (Figure 2A). Figures 3A,C illustrate the changes in the HbO across 20 s in the ipsilesional and ipsilateral M1, respectively. Higher changes in HbO across time were found when participants underwent the “complex and blurred” condition relative to other conditions across the two groups.

**Table 3:** Changes in [HbO] (ME) in left and right M1 throughout 20 s finger tapping among four conditions for patient (*n* = 15) and healthy groups (*n* = 18).

**ROIs**	**Conditions**				**Within group effect**	**Between group effect**	**Interaction effect (Group × Condition)**
	**ME-** **Clear + simple**	**ME-** **Clear + complex**	**ME-** **Blurred + simple**	**ME-** **Blurred + complex**			
					**[** ***F*** **value; partial eta squared; (** ***p-*** **value)]**		
IPSI M1							
patient	0.08 ± 0.05	0.10 ± 0.04	0.11 ± 0.05	0.15 ± 0.04	3.53; 0.10 (0.02)^*^	5.08; 0.14 (0.03)^*^	0.72; 0.02 (0.54)
Healthy	−0.05 ± 0.03	0.04 ± 0.04	0.07 ± 0.03	0.11 ± 0.04			
CONTRA M1							
patient	0.04 ± 0.02	0.08 ± 0.05	0.08 ± 0.06	0.02 ± 0.05	1.38; 0.04 (0.25)	3.34; 0.10 (0.08)	1.24; 0.04 (0.30)
Healthy	−0.07 ± 0.04	−0.05 ± 0.04	0.03 ± 0.03	0.01 ± 0.03			

*ME, mirror effect; IPSI, ipsilesional (for post-stroke patients); IPSI, right hemisphere among healthy participants; CONTRA, contralesional (for post-stroke patients); CONTRA, left hemisphere among healthy participants; M1, primary motor cortex; ROI, region of interest. Data presented in mean ± standard error of mean (μMol/L)*.

* *Signify statistical significance*.

### Mirror Effect Changes in HbO at Contralesional/Contralateral M1

The group effect on HbO was not significant [*F* = 3.34; *p* = 0.08; partial eta squared = 0.10] (Table 3; Figure 2). As expected, the effect of the task condition was not statistically significant [*F* = 1.38; *p* = 0.25; partial eta squared = 0.04]. Likewise, the group × condition effect was also not statistically significant [F = 1.24; *p* = 0.30, partial eta squared = 0.04] (Table 3; Figure 2B). Figures 3B,D illustrate the changes in the HbO across 20 s in the contralesional/contralateral M1. Relatively greater changes in the HbO were observed under the “complex and clear” and “simple and blurred” conditions (patient participants), and “simple and blurred” and “complex and blurred” conditions (healthy participants). However, these changes were not statistically significant.

### Mirror Effect Changes in HbO at Ipsilesional/Ipsilateral Precuneus

**Figure 4:**
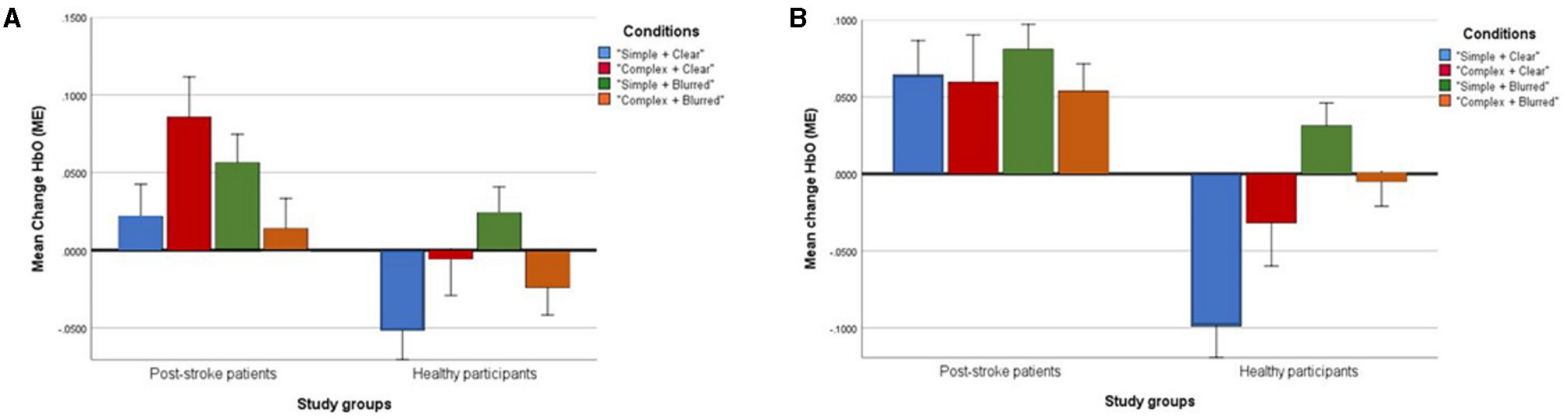
Profile plots showing changes of mean [HbO] (ME) in ipsilesional/ipsilateral Precuneus **(A)** and contralesional/contralateral Precuneus **(B)** in four experimental conditions between post-stroke patient and healthy groups (unit, μMol/L).

The group effect was statistically significant [*F* = 7.71; *p* < 0.01; partial eta squared = 0.20], with a higher mean [clear + simple: 0.06; clear + complex: 0.06; blurred + simple: 0.08 and blurred + complex: 0.05] in post-stroke patients than healthy volunteers [clear + simple: −0.10; clear + complex: −0.03; blurred + simple: 0.03 and blurred + complex: −0.01] across all experimental conditions. Conversely, a non-significant interaction of group × condition effect was found [*F* = 0.71; *p* = 0.55, partial eta squared = 0.07]. Likewise, the within condition effect was not statistically significant [*F* = 1.11; *p* = 0.36; partial eta squared = 0.10] (Table 4). Figure 4 presents a profile plot showing the HbO in the ipsilesional/ipsilateral precuneus-ROI within the experimental conditions between the groups.

**Table 4:** Changes in [HbO] (ME) in left and right Precuneus throughout 20 s finger tapping among four conditions for patient (*n* = 15) and healthy groups (*n* = 18).

**ROIs**	**Experimental conditions**				**Within group effect**	**Between group effect**	**Interaction effect** **(Group × Condition)**
	**ME- ** **Clear+ simple**	**ME-** **Clear+ complex**	**ME-** **Blurred+ simple**	**ME-** ** Blurred+ complex**			
					**[** ***F*** **value; partial eta squared; (** ***p-*** **value)]**		
IPSI Precuneus							
Patient, *n* = 15	0.06 ± 0.03	0.06 ± 0.07	0.08 ± 0.04	0.05 ± 0.04	1.11; 0.10 (0.36)	7.71; 0.20 (<0.01)^*^	0.71; 0.07 (0.55)
Healthy, *n* = 18	−0.10 ± 0.05	−0.03 ± 0.05	0.03 ± 0.03	−0.01 ± 0.03			
CONTRA Precuneus							
Post-stroke, *n* = 15	0.02 ± 0.03	0.09 ± 0.06	0.06 ± 0.04	0.01 ± 0.04	1.30; 0.12 (0.29)	3.03; 0.09 (0.09)	0.21; 0.02 (0.89)
Healthy, *n* = 18	−0.05 ± 0.05	−0.01 ± 0.05	0.02 ± 0.03	−0.02 ± 0.04			

*ME, mirror effect; IPSI, ipsilesional (for post-stroke patients); IPSI, right hemisphere among healthy participants; CONTRA, contralesional (for post-stroke patients); CONTRA, left hemisphere among healthy participants; ROI, region of interest. Data presented in mean ± standard error of mean (μMol/L)*.

* *Signify statistical significance*.

### Mirror Effect Changes in HbO at Contralesional/Contralateral Precuneus

The group effect was not statistically significant [*F* = 3.03; *p*= 0.09; partial eta squared = 0.09]. Similarly, the group × condition interaction effect [*F* = 0.21; *p* = 0.89, partial eta squared = 0.02] and within condition effect [*F* = 1.30; *p* = 0.29; partial eta squared = 0.12] were not statistically significant (Table 4). Figure 4 presents a profile plot showing the HbO in the contralesional/contralateral precuneus-ROI within the experimental conditions between the groups.

### Individual Differences in Kinesthetic Illusion

The condition effects were significant for intensity [F = 51.56; *p* < 0.01; partial eta squared = 0.63], authenticity [F = 32.50; *p* < 0.01; partial eta squared = 0.51], and perceived effort [F = 12.76; *p* < 0.01; partial eta squared = 0.29] (Table 5). However, the group and group × condition effects on all three variables were not significant. *Post-hoc* analyses revealed all pairwise comparisons of the conditions were significant in the intensity (*p* < 0.01) and authenticity of the illusion (*p* = 0.00–0.01), for all comparisons except between “simple and blurred” and “complex and blurred” conditions (intensity, *p* = 0.99; authenticity, *p* = 0.13). For the perceived effort used to generate kinesthetic sensation, *post-hoc* analyses revealed significant differences between the “simple and clear” and “simple and blurred” conditions (*p* = 0.007), and the “simple and clear” and “complex and blurred” conditions (*p* < 0.01) respectively.

**Table 5:** Differences in the intensity and authenticity of kinesthetic mirror illusion, and perceived effort of generating kinesthetic sensation across the four experimental conditions for patient (*n* = 15) and healthy groups (*n* = 18).

	**Conditions**				**Within group effect**	**Between group effect**	**Interaction effect** ** (Group × Condition)**
	**Mirror ** **Clear + simple**	**Mirror** ** Clear + complex**	**Mirror ** **Blurred + simple**	**Mirror** ** Blurred + complex**			
					**[** ***F*** **value; partial eta squared; (** ***p-*** **value)]**		
***Question 1***							
Patient	2.00 ± 0.76	2.80 ± 0.78	3.89 ± 0.74	4.00 ± 0.76	51.56; 0.63 (<0.01)^*^	0.01; 0.001 (0.93)	0.40; 0.01 (0.74)
Healthy	1.89 ± 0.68	2.94 ± 0.80	3.67 ± 0.97	4.11 ± 0.83			
***Question 2***							
Patient	2.07 ± 0.80	2.80 ± 0.56	3.47 ± 0.74	3.80 ± 0.78	32.50; 0.51 (<0.01)^*^	0.004; 0.001 (0.95)	0.31; 0.01 (0.82)
Healthy	2.06 ± 0.94	2.61 ± 0.85	3.50 ± 0.10	4.00 ± 0.69			
***Question 3***							
Patient	3.33 ± 1.29	3.00 ± 0.93	2.20 ± 0.86	1.80 ± 1.01	12.76; 0.29 (<0.01)^*^	1.35; 0.04 (0.25)	0.40; 0.01 (0.75)
Healthy	3.39 ± 1.29	3.17 ± 1.30	2.67 ± 0.77	2.28 ± 1.18			

*Data presented in mean ± standard deviation*.

*Question 1: To what extent did you feel that the movement of the displayed hand belonged to your static hand?*

*Question 2: To what extent did you feel as you were seeing your real hand?*

*Question 3: How much effort did you apply in generating the feeling of movement of your static hand behind the mirror?*

*Rating: Lower scores signify higher intensity and authenticity of kinesthetic mirror illusion (question 1 and 2) and greater perceived effort in generating the kinesthetic sensation (question 3)*.

* *Signify statistical significance*.

### Error Rates and Validity Check

The proportion of participants' correct responses as opposed to wrong responses (validity check) was found not to be significantly dependent on the test-group they belonged to across all conditions (*p* = 0.12–0.48) (Appendix D in Supplementary Material).

The condition effects on the error rates of finger tapping performed by the participants were significant [*F* = 3.45; *p* = 0.02; partial eta squared = 0.10]; but the group [*F* = 0.01; *p* = 0.94; partial eta squared = 0.001] and group × condition effects were not statistically significant [*F* = 0.18; *p* = 0.91, partial eta squared = 0.01] (Appendix E in Supplementary Material). Nevertheless, greater finger tapping complexity and increased blurriness of mirror image resulted in an increase in the error rates. *Post-hoc* analyses revealed significantly higher error rates under the “simple and blurred” than “simple and clear” conditions (*p* = 0.03).

## Discussion

This study explored the effects of task complexity and image clarity on modulating the neural activities in the motor cortex and precuneus associated with mirror therapy. This is the first study manipulating both factors among a patient population. The findings indicated the influence of both task complexity and image clarity on higher activities in the ipsilateral M1. The patient participants showed significantly stronger activities in the motor cortex across all test conditions when compared to healthy adults. The stronger neural activities observed among the patient participants compared with the healthy controls highlight their differential neural activities, likely due to deficiencies in inter-hemispheric activation balance and associated changes resulting from brain lesions after stroke (14). The effects were strongest in the ipsilateral and ipsilesional hemisphere for the “complex and burred” condition in both groups. However, such effects were not apparent in the contralateral/contralesional hemisphere. Therefore, this finding highlights the extent of top-down cognitive influence on the motor network in the mirror therapy paradigm and the effect of manipulation on cognitive load (31–33).

This study's main finding is an increase of blood hemoglobin concentration (HbO) in both groups of participants when performing complex vs. simple finger tapping movements and when viewing blurred vs. clear images. This finding indicates stronger activation in the ipsilateral M1 when motor sequences were more complex and when motor-related images were blurred and hence required more effort to visualize.

A previous review attributed the increase of ipsilateral M1 involvement to an increase of top-down cognitive influences (8). Our findings relating to an increase of ipsilateral M1 activation are consistent with another study using the mirror-induced visual illusion (MVI) paradigm, which reported that a decrease in the speed of mirror visual feedback resulted in stronger amplitudes of the motor evoked potential at the M1 sites (64). Similarly, a 2-s delay in mirror visual feedback increased event-related desynchronization in the ipsilateral M1 (65). These two studies, together with a few other studies, concluded that M1 initiated top-down influence was modulated by conflicting perceptual processes between the kinesthetic experiences (i.e., movements of the fingers) and the observed images of movement (8, 9, 64, 65). Exposure to incongruent information causes the human brain to activate processes to resolve conflicts between multiple senses, which results in different modulatory effects on the motor and perceptual processes (3, 64). Incorporating task complexity and image blurriness manipulations might have enhanced perceptual conflict, thereby leading to an increased effect on the motor system. A similar influence on the contralateral motor system has not been found, likely due to the effect of movement execution on the contralateral hemisphere. Crucially, increased neural activity in the motor area of the ipsilesional hemisphere in post-stroke patients is regarded as a marker of functional recovery (14). Kinesthetic motor imagery shares neural substrates and associated mental processes with mirror therapy (10). Menatal rehearsal of complex finger tapping movement, using the kinaesthetic modality resulted in increased activity in the premotor cortex, posterior parietal, and cerebellar region relative to simple finger movement imagery (19). These brain areas are strongly associated with motor control and cognitive facets of motor processes, including movement selection, preparation, and motor imagery (31, 66). Therefore, such an increase in activities further illustrates the similarities in neural responses between movement execution and motor imagery (67). This finding serves as further evidence of the usefulness of mental simulation of movement in the absence of movement execution.

In our study, increased activation in the ipsilesional and ipsilateral M1 was unique to the most challenging “complex and blurred” condition. These findings are inconsistent with those reported by Bai et al. (62), which indicated that activation in the M1 based on fNIRS during a MVI Purdue pegboard assembly task was comparable to that captured during the same MVI task without using the Purdue pegboard. The inconsistency may be attributed to differences in the task designs and instructions between the two studies. The instructions given to our participants were to visualize and feel the static hand (placed behind the mirror) according to the mirror images. In contrast, no explicit instructions were given in Bai et al.'s study. Clearer instructions would have ensured that participants experienced perceptual conflicts and hence shown increased M1 activity. This argument concurs with observations made in other studies among post-stroke patients and healthy participants where clearer imagery instructions resulted in significant activation of the ipsilateral motor system using the mirror therapy paradigm (24, 40).

In contrast to the M1, the “complex and blurred” condition did not produce a similar effect of increased activation in the precuneus in both the patient and healthy groups. Trends of increases in neural activities in the ipsilesional/ipsilateral precuneus with increased finger tapping complexity and blurriness of mirror images were found. However, these trends did not reach statistical significance, which could have been due to the small sample sizes or inadequate effect sizes of the different conditions. The precuneus is a key neural substrate, mediating visualization and processing motor images of oneself in the MVI paradigm (8, 55, 56, 68). Increases in precuneus activities have been associated with functional regains resulting from MVI training in post-stroke patients (56). Compared with the M1, the precuneus has diverse roles that subserve a wide range of cognitive and motor-related processes (69), including visuospatial perception, body image representation, self-processing operation, and retrieval of stored visuospatial percepts during mental rehearsal and memory recall (8, 69–71). Because of its diverse roles, activities in the precuneus during the MVI paradigm were found to be more prone to be confounded by the design and logistics of a study. For instance, different results were obtained when mirror images were projected on the anterior plane of the viewer, as in virtual reality mirror box or real-time video recording, than those projected at mid-sagittal positions as in conventional mirror box (8, 10). Further studies using larger sample sizes and well-designed tasks are called for to refine the role of the visuo-perceptual processes, as reflected in precuneus activity, when engaging in the MVI paradigm.

Participants in the patient group showed significantly higher activity in the ipsilesional M1 and precuneus across all experimental conditions relative to those in the healthy group. The non-significant group × condition finding is worth further discussion. First, the significant between-group differences do not concur with those reported by Kang et al. (40), who found using transcranial magnetic stimulation (TMS) that healthy participants had significantly higher MEP amplitude than post-stroke patients in the MVI paradigm, suggesting higher motor facilitation among the healthy volunteers. On the other hand, Wang et al. (72), using functional magnetic resonance imaging (fMRI), reported comparable precuneus activations between post-stroke patients and healthy volunteers. Similar findings of comparable neural responses to MVI between post-stroke patients and healthy volunteers have been reported elsewhere (8). The discrepant findings reported in our study could be associated with deficits of the ipsilesional hemisphere among post-stroke patients (14) in comparison with a relatively balanced level of neural activity found in the bilateral hemispheres in the healthy human brain. As such, the neural response to the MVI paradigm might have resulted in marked changes in the neural system among the post-stroke patients. Furthermore, variations in the complexity and nature of tasks between our study and those reported above may explain the conflicting results. Similarly, unilateral finger movement is associated with minimal transcallosal transfer of neural activity to the ipsilateral hemisphere in the movement execution paradigm (73). To control for the effect of movement execution on the selected ROIs in our study, the mirror effect (ME) was analyzed by subtracting the change in HbO concentration of a control condition from the corresponding experimental condition (44). Kang et al.'s (40) study utilized a baseline/rest condition to determine the change of neural activity in the ipsilesional/ipsilateral M1-ROI. Therefore, the ME in their study might have been contaminated by the influence of the minimal transcallosal transfer; discrepancies in the data analysis process might have also resulted in the inconsistent findings. Greater increase in the neural activity among post-stroke patients and a lack of similar effect among healthy volunteers due to the introduction of an MVI paradigm further explains its facilitatory effect on the impaired neural system (43).

### Study Strengths and Limitations

This study has a number of strengths. Firstly, the study findings provided a deeper understanding of the ME using a conventional mirror therapy setup commonly used in clinical settings, which allows easy transfer of information into clinical practice. Secondly, using fNIRS allowed an assessment of neurovascular changes in the ROIs without requiring participants to lie within a scanner, as in the case of fMRI. Lastly, we controlled for the movement execution effect on ipsilesional/ipsilateral M1 by providing unique control conditions for each of the corresponding experimental condition.

Nonetheless, the study has a few limitations. Firstly, the study design was unable to check whether participants invested full effort in viewing the mirror images of the moving hand, despite providing clear instructions and practice before the experiment. Future studies can employ eye-tracking to ensure participants' concentration and involvement during the task. Secondly, the post-stroke patients lived in the community. The research team was able to obtain detailed information on the lesion site for only five participants, which did not allow meaningful exploration of how lesion sites modulate M1 activation. Thirdly, the combination of both right- and left-brain lesions, and not matching the patient group with the control group in terms of hand use might have confounded the fNIRS results. Future studies are to place control for the lesioned hemisphere and match the hand use between the experimental and control groups. The experimental task took more than 1 h to complete. Despite intermittent rest periods, participants could have experienced fatigue during the task, which would have influenced the results of the conditions toward the end of the assessment. However, the presentation of the experimental and control conditions was pseudo-randomized using E-prime software to control for possible order effect. Thus, future study using a larger sample size and restricting the post-stroke patients by the site of lesion is warranted. Lastly, the design of the complex task used in this study might not have pitched at a difficulty level which had been sufficient to trigger differential neural activities in both the post-stroke patient and control groups. This resulted in the non-significant between-group and condition interaction effects. Future study is to test the difficulty level of the complex task which can accommodate the potentially different levels of abilities of the patient and healthy groups.

## Conclusion

Study findings indicated that increasing the complexity of finger movements and blurring their mirror images increased activities in the motor cortex in post-stroke patients. Future studies should explore how this neural process is part of the mechanism underlying the positive treatment effects of mirror therapy for post-stroke patients.

## Data Availability Statement

The raw data supporting the conclusions of this article will be made available by the authors, without undue reservation.

## Ethics Statement

The studies involving human participants were reviewed and approved by Human subjects' ethics sub-committee of the Hong Kong Polytechnic University (HSEARS20190129002). The patients/participants provided their written informed consent to participate in this study.

## Author Contributions

UMB: conceptualization, methodology, formal analysis, data curation, writing - original draft, and visualization. SJW: conceptualization, methodology, resources, writing - review and editing, and supervision. CC: conceptualization, methodology, writing - review and editing, and supervision. All authors contributed to the article and approved the submitted version.

## Funding

The work of UMB was supported by PhD studentship of The Hong Kong Polytechnic University.

## Conflict of Interest

The authors declare that the research was conducted in the absence of any commercial or financial relationships that could be construed as a potential conflict of interest.

## Publisher's Note

All claims expressed in this article are solely those of the authors and do not necessarily represent those of their affiliated organizations, or those of the publisher, the editors and the reviewers. Any product that may be evaluated in this article, or claim that may be made by its manufacturer, is not guaranteed or endorsed by the publisher.
