# Supplementary material for: Task Complexity and Image Clarity Facilitate Motor and Visuo-Motor Activities in Mirror Therapy in Post-stroke Patients
Source: Front Neurol. 2021 Sep 22;12:722846. doi: 10.3389/fneur.2021.722846 (PMC8493295; doi:10.3389/fneur.2021.722846)
Supplement: Supplementary file 1 [file Data_Sheet_1.docx]

**Appendix A:** Rating of vividness of kinesthetic mirror illusion and internal generation of movement sensation questionnaire

***Instructions:***

- Please, use the 5-point rating scale below to answer **question 1 and 2.**

1. Perfectly clear and as vivid as normally felt movement
2. Clear and reasonably vivid
3. Moderately clear and vivid
4. Vague and dim
5. Not felt at all

- Please, use the 5-point rating scale below to **answer question 3.**

1= “very much effort” to 5= “no effort at all”.

***Questions:***

- Question 1: To what extent did you feel that the movement of the displayed hand belonged to your affected/left hand?
- Question 2: To what extent did you feel as you were seeing your real hand?
- Question 3: How much effort did you apply in generating the feeling of movement of your affected/left hand?

Thank you

**Appendix B:** Socio-demographic characteristics of the healthy participants (n = 18)

| Participants | Age | Sex | Educational level | Use of visual aid |
| --- | --- | --- | --- | --- |
| 1 | 67 | F | Primary | No |
| 2 | 58 | F | Primary | No |
| 3 | 70 | M | Secondary | Yes |
| 4 | 56 | F | Secondary | No |
| 5 | 71 | M | Primary | Yes |
| 6 | 60 | F | Secondary | Yes |
| 7 | 54 | F | Secondary | No |
| 8 | 59 | M | Degree | Yes |
| 9 | 58 | F | Secondary | No |
| 10 | 57 | M | Diploma | Yes |
| 11 | 56 | F | Degree | Yes |
| 12 | 53 | M | Secondary | No |
| 13 | 64 | M | Secondary | Yes |
| 14 | 57 | M | Diploma | Yes |
| 15 | 51 | M | Masters | No |
| 16 | 75 | F | Secondary | Yes |
| 17 | 75 | M | Secondary | Yes |
| 18 | 59 | M | degree | Yes |
|  | 61.1±7.4 | M: 55.6% |  | Yes:61.1% |

*Hand dominance:* All the healthy participants were right handers.

**Appendix C:** HbO change in M1 and precuneus region of interest across experimental conditions

|  | **Mean change of HbO in M1-ROI across experimental and control conditions (µMol/L)** | | | | | | | | | | | | | | | |
| --- | --- | --- | --- | --- | --- | --- | --- | --- | --- | --- | --- | --- | --- | --- | --- | --- |
|  | **CLS** | | | | **CLC** | | | | **BS** | | | | **BC** | | | |
|  | ***EXP-***IPSI | ***CON-***IPSI | ***EXP-***CLT | ***CON-***CLT | ***EXP-***  IPSI | ***CON-***  IPSI | ***EXP-***  CLT | ***CON-***  CLT | ***EXP-***IPSI | ***CON-***IPSI | ***EXP-***CLT | ***CON-***CLT | ***EXP-***IPSI | ***CON-***IPSI | ***EXP-***CLT | ***CON-***CLT |
| **STROKE SURVIVORS (N=15)** | | | | | | | | | | | | | | | | |
|  | .1159  (.04) | .0220  (.04) | .1123  (.04) | .0773  (.04) | .10223  (.05) | -.0021  (.05) | .1409  (.07) | .0585  (.06) | 01139  (.03) | .0024  (.05) | 1.456  (.05) | .0662  (.06) | .1470  (.05) | -.0061  (.05) | .1382  (.05) | .1211  (.05) |
| **HEALTHY VOLUNTEERS (N=18)** | | | | | | | | | | | | | | | | |
|  | .0677  (.02) | .1158  (.02) | .0670  (.03) | .1326  (.03) | .1143  (.04) | .0789  (.04) | .0935  (.05) | .1437  (.05) | .1120  (.02) | .0441  (.04) | .1340  (.02) | .1015  (0.4) | .2085  (.03) | .0936  (.03) | .1680  (.04) | .1589  (.04) |
|  | **Mean change of HbO in Precuneus-ROI across experimental and control conditions (µMol/L)** | | | | | | | | | | | | | | | |
| **STROKE SURVIVORS (N=15)** | | | | | | | | | | | | | | | | |
|  | .0604  (.04) | .0078  (.03) | .0732  (.04) | .0517  (.05) | .1163  (.05) | .0568  (.08) | .1647  (.04) | .0791  (.06) | .0965  (.03) | .0156  (.05) | .0954  (.04) | .0391  (.03) | .0728  (.04) | .0191  (.03) | .1112  (.04) | .0974  (.05) |
| **HEALTHY VOLUNTEERS (N=18)** | | | | | | | | | | | | | | | | |
|  | .1025  (.03) | .2014  (.04) | .1227  (.03) | .1741  (.04) | .1386  (.05) | .1703  (.04) | .1397  (.04) | .1452  (.04) | .1269  (.03) | .0958  (.03) | .1089  (.03) | .0849  (.04) | .2000  (.04) | .2048  (.03) | .1791  (.04) | .2030  (.03) |

*Abbreviations:* HbO, oxygenated haemoglobin change; M1, primary motor cortex; ROI, region of interest; CLS, clear+simple; CLC, clear+complex; BS, blurred+simple; BC, blurred+complex; EXP, experimental condition; CON, control condition; IPSI, ipsilateral hemisphere; CLT, contralateral hemisphere. Mean (standard error of mean).

**Appendix D:** Validity check of task participation across the four experimental conditions for patient (n=15) and healthy groups (n=18)

| **Experimental condition** | **Responses** | **Post-stroke patients (N=15)** | **Healthy participants**  **(N= 18)** | **Total** | **Chi-square statistics** |
| --- | --- | --- | --- | --- | --- |
| Mirror Clear + Simple | Right | 11 | 15 | 26 | X^2^ = 0.49, *p* = 0.48 |
|  | Wrong | 4 | 3 | 7 |  |
| Mirror Clear + Complex | Right | 10 | 16 | 26 | X^2^ = 2.42, *p* = 0.12 |
|  | Wrong | 5 | 2 | 7 |  |
| Mirror Blurred + Simple | Right | 10 | 14 | 24 | X^2^ = 0.51, *p* = 0.48 |
|  | Wrong | 5 | 4 | 9 |  |
| Mirror Blurred + Complex | Right | 7 | 11 | 18 | X^2^ = 0.69, *p =* 0.41 |
|  | Wrong | 8 | 7 | 15 |  |

**Appendix E:** Finger tapping error rates across the four experimental conditions for patient (n=15) and healthy groups (n=18)

|  | Conditions | | | | Within Group effect | Between Group effect | Interaction effect  (Group ×Condition) |
| --- | --- | --- | --- | --- | --- | --- | --- |
|  | Mirror Clear + Simple | Mirror Clear + Complex | Mirror Blurred + Simple | Mirror Blurred + Complex |  |  |  |
|  |  |  |  |  | [*F* value; partial eta squared; (*p-*value)] | | |
| Error rates    Patient | 0.03±0.04 | 0.05±0.07 | 0.05±0.05 | 0.06±0.06 | 3.45; 0.10 (0.02)* | 0.01; 0.001 (0.94) | 0.18; 0.01 (0.91) |
| Healthy | 0.03±0.05 | 0.04±0.06 | 0.06±0.08 | 0.06±0.08 |  |  |  |

Data presented in mean±standard deviation
